# Supplementary material for: Are carbon emissions trading and green financial instruments synergistic? -Comprehensive quantitative research based on content analysis
Source: PLoS One. 2024 Mar 7;19(3):e0298601. doi: 10.1371/journal.pone.0298601 (PMC10919601; doi:10.1371/journal.pone.0298601)
Supplement: S1 Data — (ZIP) [file pone.0298601.s001.zip › Data/地级市Z_F工作报告.zip!/╣π╢1⁄2/╒╪╟∞╩╨/└┤╫╘╣1⁄2╓┌║┼í╛┬φ┐╦╩2╛▌═°í┐_2018.docx]

各位代表：

　　我代表肇庆市人民政府向大会作工作报告，请予审议，并请政协委员和其他列席人员提出意见。

　　2017年工作回顾

　　过去一年，是新一届政府的开局之年。市政府坚持以习近平新时代中国特色社会主义思想为指导，全面贯彻落实党的十八大、十八届历次全会精神，深入学习贯彻党的十九大精神，以习近平总书记对广东作出的重要指示批示精神为统领，按照省、市第十二次党代会和市十三届人大一次会议的决策部署，在市人大和市政协的监督支持下，坚持稳中求进工作总基调，牢固树立新发展理念，坚持解放思想、东融西联、产业强市、实干兴肇，紧紧围绕建设枢纽门户城市目标，聚焦“366”和“1133”两大工程，突出“三大建设”，打造“三大品牌”，强化“三大动力”，扎实做好稳增长、促改革、调结构、惠民生、防风险各项工作，推动经济社会保持平稳健康发展态势，加快建设成为广东面向大西南枢纽门户城市的良好势头日趋显现。

　　一、坚持稳中求进工作总基调，经济运行企稳向好

　　主要经济指标逐渐回暖。采取有力措施应对经济下行压力，主要经济指标稳中有进，财政质量达到“十一五”以来最好水平。预计（下同），全年完成地区生产总值2190亿元，比上年增长（以下简称“增长”）5%，一二三产业增加值分别增长4.7%、4.5%、6.2%。人均生产总值提升到53400元，增长4%。规模以上工业增加值、固定资产投资、社会消费品零售总额分别增长5%、9%、10.5%。一般公共预算收入94.85亿元，增长5.8%，增速实现由负转正，非税占比下降到27.9%。全体居民人均可支配收入22500元，实际增长7.8%。

　　要素投入拉动经济作用充分发挥。以大融资大投入推动大开发大建设，拉动经济发展成效明显。争取上级财政补助资金138亿元，政府性投资项目获批融资444亿元。全市投资超亿元项目263个，完成投资560亿元，增长65.3%。基础设施和高技术产业投资分别增长57%、38.7%。超额完成省、市重点项目年度投资计划，100项市重点项目全年完成投资545亿元，其中35项省重点项目完成投资计划152.7%，完成率居全省前列。

　　存量资源配置进一步优化。抓好“三去一降一补”，进一步激发市场活力。支持民营企业发展，全年为企业减负约49亿元，新增新三板挂牌企业、区域股权交易市场注册挂牌企业各5家。9家钢铁落后产能企业全部关停，54家国有“僵尸企业”出清重组。获批新增建设用地1.21万亩，盘活闲置土地3776亩，完成“三旧”改造4569亩。

　　发展活力进一步增强。大力推进商事制度改革，实行企业开办“多证合一”服务模式，开办企业便利度跃居全省第一梯队。新登记各类企业增长43.5%，全市实有各类商事主体总量首次超过20万户。创新户籍制度改革，实现“零门槛”入户。成为全省率先实现国际贸易“单一窗口”口岸全覆盖地级市之一，通关便利化进一步提升。成功举办粤港澳大湾区发展高峰论坛，融入广佛肇清云韶经济圈步伐不断加快，承接珠三角地区产业梯度转移项目54个，计划总投资273亿元。

　　风险防控能力进一步提高。坚决贯彻落实重点防控化解金融风险各项要求，积极消化存量债务，全市预警地区债务风险水平总体下降，预计市本级可退出预警名单。坚持有序出让土地，加快建立租购并举的住房制度，成为全国首批12个开展住房租赁试点的城市之一，商品住房库存消化周期为6个月。

　　二、全力推进“366”工程，主导产业发展势头良好

　　工业发展“366”工程开局顺利。新能源汽车、先进装备制造、节能环保三大主导产业占比逐渐提升，发展速度明显加快。新增年主营业务收入超亿元企业50家，预计新增入规模库企业150家，5家企业进入广东企业500强。新能源汽车产业率先发展，小鹏汽车超百亿项目签约动工，中电首批新能源汽车整车下线并交付使用，“肇庆金秋”4个新能源汽车项目总投资超180亿元。先进装备制造业新引进、新开工、新投产超亿元项目分别为22个、18个、14个。高端膜材料生产基地等重大环保项目落户。

　　“招商引资年”成效明显。创新招商引资模式，招商引资精准性和效益进一步提升。京东华南智谷、新兴际华华南“安全谷”等一批大项目相继落户。“肇庆金秋”签约项目78宗，投资总额980亿元，工业项目占比提升至78.2%，产业类项目投资规模和项目质量取得新突破。全年引进合同项目198宗，其中超20亿元项目15宗。

　　“工业园区建设年”成效初现。园区建设有力推进，承载和集聚能力不断增强。新增园区建设用地规模4.35万亩，新开发及盘活用地2.04万亩，启动建设通用厂房222万平方米、建成100万平方米。新增落地开工项目96个，完成投资362亿元。投产项目117个，完成投资127亿元。

　　三、大力实施“1133”工程，发展新动能进一步增强

　　创新驱动发展战略取得新成效。全面启动国家创新型城市和知识产权示范城市创建工作，制订市高新技术企业、新型研发机构认定和扶持暂行办法，设立10亿元产业投资引导基金，研发投入占GDP比重提高到1.5%左右。新增国家高新技术企业107家，高技术产业增加值增长14.4%，国家级科技企业孵化器实现“零”的突破，新增2家国家级众创空间，省级创新平台、科技企业孵化器分别新增54家、12家，实现科技企业孵化器县（市、区）全覆盖和大型工业企业研发机构全覆盖，发明专利申请量、拥有量分别增长101%、25%。

　　高新区国家自创区建设取得新进展。由国家知识产权试点园区升级为示范园区。设立25亿元新兴产业投资基金支持创新发展，启动编制高新区220平方公里扩容规划，引进广东国腾量子科技等一流科研实体，以柔性引才方式引进院士9人。高新技术企业占园区规上企业比例首次突破50%，各项科技创新指标占全市比例提升至三分之一以上。

　　高层次人才引育迈上新台阶。高水平建成鼎湖高层次人才“双创园”、高新区创新创业科学园等人才创新创业平台，实现“百千万人才工程”国家级人选、“珠江人才计划”创新创业团队和领军人才“零”的突破，人才引进数量和质量创新高。累计引进培育国家级人才12人，引进西江创新创业团队6个、领军人才3名。

　　四、紧抓城市建设“火车头”，城市发展格局不断优化

　　新区崛起加快城市东进发展。启动编制新区480平方公里扩容规划，肇庆东站站前综合体主体结构顺利封顶，东进大道一二期主体工程基本完工、三期工程动工建设，城市东进的发展趋势更加明显。全面推动地下综合管廊和六大水系基础设施建设，39个重点项目完成年度投资135亿元，医院、学校、文化、体育、商务办公等城市元素密集布点，核心区基本实现基础设施、土地收储全覆盖，起步区城市框架逐步形成。新引进龙光集团广佛肇总部、招商局港口控股港航产业等项目18个，协议和签约合同总额1087亿元，集聚了京东云华南总部等近400亿元的数字经济项目。粤港澳大湾区生态科技产业园动工建设，孵化创新园加快推进。

　　府城复兴提升城市品质。聚焦府城复兴实施城区品质提升工程，着力改变城区老旧、交通拥挤、格局狭窄现状，古城焕发出新活力。完成府城保护与复兴项目一期工程征拆和三个古城门考古发掘工作，加快推进古城墙修缮保护、城墙公园、包公府衙、两广总督府等项目，基本再现千年府城轮廓。深得民心的端州城区50条“瓶颈路”改造有16条建成通车，北岭路网升级改造工程基本完成，改善了城区交通，提升了城区品质。

　　交通大会战提高外联内通水平。完成交通基础设施建设投资132亿元。汕昆、汕湛、怀阳高速肇庆段以及广佛肇高速广州石井至肇庆大旺段按计划推进，肇明、佛肇高速纳入省高速公路网规划。西江（界首至肇庆）航道扩能升级工程基本完成，全市港口货物吞吐量增长21%。阅江大桥建成通车，肇庆大桥扩建工程加快推进。

　　五、坚持农业农村优先发展，“三农”工作扎实推进

　　脱贫攻坚取得决定性进展。抓好脱贫攻坚“八项工程”，打好政策组合拳，强化“两不愁、三保障”政策落实，两年累计投入扶贫资金9.75亿元，全年再实现4万贫困人口预脱贫。

　　农业产业化步伐加快。新增省级农业龙头企业3家、农民合作示范社18家、家庭示范农场117家，新增省级农业名牌产品9个，新培育12个农产品电子商务产业基地。广宁县建成粤西地区最大油茶深加工项目，德庆县成为首批国家农村产业融合发展示范园创建单位，封开县杏花鸡等品牌农业发展壮大，怀集县食品饮料产业集聚效应明显。

　　新农村建设稳步推进。补齐农村人居环境和基础设施建设短板。支持山区板块各项建设和民生等补助4.64亿元，比上年提高11.3%。完成农村公路硬底化316公里，新增425条村通自来水、惠及120.9万人，动工改造农村危房8300户，实现农村基础金融服务“村村通”和行政村通光纤全覆盖。启动四会市创建省全域推进社会主义新农村建设示范县工作，新增省卫生村280个，绿化美化乡村117个。稳步推进农村土地三权分置，农村土地承包经营权确权颁证率达到94.8%，进度居全省前列。全面完成111个省定贫困村规划编制，913个20户以上自然村完成“三清三拆三整治”。

　　六、万众同心齐“创文”，社会民生事业全面发展

　　“创文”攻坚得到群众认可和各界好评。发动全市力量吹响“创文”集结号，积极倡导社会主义核心价值观，文明程度大幅提升。11项专项提升行动成效显著，投入16亿元办好“万件民生微实事”，完成老旧小区改造156个、农贸市场升级改造78个。全市有6项10个单位获全国文明表彰、5项15个单位获全省文明表彰，四会市成功创建全国县级文明城市，德庆县获评“广东省县级文明城市”。“创文”凝聚了全市人民共创共建的汗水和心血，得到了全市人民高度支持和认同，彰显了全市上下万众一心追求新时代新肇庆的精神风貌。

　　打好污染防治攻坚战推进“绿富同兴”。扎实推进中央环保督察反馈问题整改，圆满完成省环保督察迎检工作，全面启动国家生态文明建设示范市创建工作，推动环境质量持续好转。强化大气污染防治，全市空气质量优良天数比例达93.4%，城区空气质量综合指数在全省排名上升1位。全面落实“河（湖）长制”，制定西江水质保护规划和负面清单，6个县（市、区）整县推进新一轮污水处理设施建设，独水河高新区段完成生态修复，羚山涌和石咀涌水质明显改善，全市地表水省考断面水质优良率达91.7%。打击非法采（运）河砂行为持续深入，违法禽畜养殖有效整治。深入推进绿化肇庆大行动，完成造林16.92万亩，新增森林公园13个。

　　民生保障改善有新进展。财政投入民生类支出198.24亿元，占一般公共预算支出的72.96%。如期完成省、市十件民生实事（详见附件一）。城镇登记失业率控制在3%的目标以内。全部县（市、区）通过“广东省推进教育现代化先进县”督导验收，新增中小学位9040个，省市共建肇庆学院启动实施，广东工商职业学院“升本”通过省教育厅验收。公立医院综合改革实现全覆盖，完成18间乡镇卫生院标准化和335间村卫生站规范化建设。实现企业职工基本养老保险省级统筹和异地就医联网结算。城乡社区养老服务设施实现全覆盖。100%安置退役士兵和随军家属。建成公共租赁住房、棚户区改造住房5093套（户）。第十五届省运会筹备工作全面有序开展，34个场馆建设改造进展顺利。市文化发展中心主体工程完工。成功举办肇庆国际半程马拉松赛、中国童声合唱节、万人书写大会等重大文体活动。质量强市、食品药品安全监管深入推进，各类生产安全事故同比下降23.9%，社会治安形势总体平稳向好。成功抵御台风“天鸽”等自然灾害。统计、民族、宗教、革命老区、外事、侨务、港澳、对台、人防、国防动员、地方志、档案、气象、水文、地震、妇女儿童、青少年、残疾人、慈善等工作取得新进步。

　　七、坚定不移加强政府自身建设，运行效能进一步提升

　　深入开展“解放思想，实干兴肇”教育实践活动，强化政府部门“统筹、协调、指导、监督”意识，着力破解干部队伍思想不解放、作风不转变、为官不作为等庸政懒政怠政现象。坚持向市人大及其常委会报告工作，向市政协通报情况，自觉接受监督，按期办结人大代表建议129件、政协提案146件。组织和参与起草市人大地方性法规草案3件，颁布实施市政府规章2件，行政复议案件依期结案率100%。全面落实行政执法责任制。建成投资项目在线审批监管平台，行政审批事项网上全流程办理率和办结率分别为99.8%和92.4%，142项事项实现“零跑动”办理。完成市属事业单位和国企公车改革。国家、省交办信访积案结案率100%。强化行政监察、审计监督，党风廉政建设扎实有效。

　　各位代表，以上成绩的取得，是省委、省政府和市委正确领导的结果，是市人大、市政协监督支持的结果，是全市人民和社会各界团结奋斗、开拓进取的结果。我代表市人民政府，向全市人民，向各位人大代表、政协委员，向各民主党派、各人民团体、各界人士，向驻肇部队和各级驻肇单位，向老领导、老同志，向所有关心支持参与肇庆建设发展的海内外各界人士，致以崇高的敬意和衷心的感谢！

　　对照党的十九大精神和中央的部署，按照高质量发展的要求，全市经济社会发展还存在不少困难和问题，比如：东南板块与西北板块协调发展的任务艰巨；经济总量偏小，GDP、工业等部分主要经济指标未能完成全年预期目标任务，外贸进出口总额、实际吸收外资明显下滑；高新技术企业储备量、高新技术产业产值占比偏低，产业项目落地转化率还不高；城镇化水平亟待提升，新农村建设和脱贫攻坚任务艰巨；“创文”需要进一步向纵深推进；空气质量综合治理形势依然严峻，教育、卫生、交通等公共服务水平有待提高；政府行政服务效能还有待提升，等等。这些问题是我市经济社会发展不平衡不充分的具体体现，我们将以高度的责任感和紧迫感，切实解决好影响全市经济社会发展的困难和问题。

　　2018年工作安排

　　2018年是全面贯彻党的十九大精神的开局之年，是决胜全面建成小康社会、实施“十三五”规划承上启下的关键一年。中央经济工作会议明确提出了习近平新时代中国特色社会主义经济思想，这是指导我们做好经济工作的根本遵循。省委十二届三次全会坚持以习近平新时代中国特色社会主义思想统领广东一切工作，为广东奋力开创改革发展新局作出了全面部署。今年是改革开放40周年，我们要以解放思想、实干兴肇的改革精神推动肇庆加快发展、高质量发展，使解放思想成为新时代新肇庆的新动力，实干兴肇成为抓落实促发展的新作风。

　　政府工作的总体要求是：以习近平新时代中国特色社会主义思想为指导，全面贯彻党的十九大精神，按照省委、省政府和市委的决策部署，坚持稳中求进工作总基调，坚持新发展理念，坚持高质量发展，统筹推进“五位一体”总体布局，协调推进“四个全面”战略布局，以供给侧结构性改革为主线，解放思想、东融西联、产业强市、实干兴肇，坚决打好决胜全面建成小康社会三大攻坚战，牢牢把握粤港澳大湾区发展机遇，持之以恒推进工业发展“366”和创新驱动发展“1133”两大工程，持之以恒推进全国文明城市、国家创新型城市和国家生态文明建设示范市三大创建，持之以恒解决区域发展不协调问题，实施乡村振兴战略，建设美丽肇庆，推动肇庆经济社会持续健康向上发展，加快建设广东面向大西南枢纽门户城市。

　　全市经济社会发展主要预期目标是：地区生产总值增长6.5%左右，人均生产总值增长5.5%，规模以上工业增加值增长7.7%，固定资产投资增长10%，社会消费品零售总额增长9.5%，外贸进出口总额增长1%，实际吸收外资增长10%，一般公共预算收入增长10%，R&D占GDP比重提高到1.8%左右，全体居民人均可支配收入增长与经济增长同步，居民消费价格指数涨幅控制在3%左右，城镇登记失业率控制在3%以内，节能减排和空气质量改善目标控制在省下达的计划以内。

　　重点抓好以下九个方面的工作：

　　一、深入推进“366”工程，促进经济高质量发展

　　着力深化供给侧结构性改革。重点在“破”“立”“降”上下功夫，不断夯实产业强市基础。大力破除无效供给，加大七大传统低效行业退出和整治提升力度，对产值低、能耗高、污染重的企业依法依规淘汰，印染、电镀企业实施入园管理，陶瓷行业实施综合治理和全流程监管，全面完成国有“僵尸企业”出清重组。加大力度扶持现有企业做大做强，全面落实国家和省市降成本扶实体政策，实施“一企一策”，健全中小微企业服务体系，着力帮助企业解决融资难、融资贵等问题，及时跟踪协调解决企业增资扩产、技术改造等过程中遇到的用地、用工等瓶颈问题，扶持一批龙头骨干企业向50亿、100亿规模发展壮大。实施新一轮工业企业技术改造三年行动计划，年内引导200家规上工业企业开展技术改造。支持建筑材料、金属制品等传统优势产业转型升级。

　　着力振兴实体经济。必须咬定青山不放松，全力抓好“366”工程。抓出“工业园区建设年”新成效。进一步解决园区建设土地、资金等要素保障问题，确保新开发及盘活用地1.5万亩以上，完成200万平方米工业通用厂房建设任务，其中科技孵化器厂房80万平方米。实现园区规上工业增加值增长10%。加快推动20万平方米的肇庆新区孵化创新园项目建设，为创新型企业进驻提供发展空间。西北板块重点抓好低效园区整改转化和“腾笼换鸟”，逐渐形成以产业聚集为基础的园区发展格局。开创“招商引资年”新局面。瞄准深圳、北京等重点区域精准招商，着力引进数字经济等新产业新业态。做大做强招商平台，积极构建“政府+商协会”“政府+科研机构”等联合招商模式，进一步发挥中国环保上市公司峰会和中国民商理事会年会的作用，推动招商引资向招商引资与选商引智并举转变。建立大项目投资者“绿卡”制度。力争引进投资超百亿项目3个，超10亿项目15个。迈出“项目落地年”新步伐。实行重点项目“首席服务官”、“会商”和“督报”制度，对签约投资5亿元以上产业项目和1亿元以上工业项目全部实施市、县领导挂点，着力解决项目落地的土地保障、项目审批和要素协调等问题，切实提高项目资金到位率、动工率和投产率。在每个县（市、区）年初召开项目落地现场会，年中召开专题招商会，年底召开经济督导分析会。全面落实工业优先用地，着力推动中电科肇庆产业园、新兴际华华南“安全谷”等龙头项目动工建设，力争新能源汽车、先进装备制造、节能环保三大产业产值分别达到370亿元、530亿元、350亿元。新增年主营业务收入超亿元工业企业130家左右、规上工业企业150家。

　　着力释放消费潜力。依托京东等电商龙头企业构建智慧物流体系，着力培育中高端消费、共享经济等内需增长点。高标准推进全域旅游示范市建设，全力推动星湖景区创5A，办好肇庆国际马拉松赛、粤港澳旅游美食节等品牌活动，加快建设华侨城卡乐文化旅游科技产业小镇等项目，有效刺激经济持续向好。建立多主体供给、多渠道保障、租购并举住房制度，构建住房租赁信息平台，合理调控库存，促进房地产市场健康发展。

　　着力防范化解金融风险。坚持“善用是能力、管控是责任、增值是水平、能还是底线”，加强重点领域风险防控，持续发挥金融撬动作用，用发展成果来增强防范和化解风险的能力和底气，牢牢守住不发生系统性区域性金融风险的底线。成立产业投资基金，建设社会征信和金融服务一体化系统，完成肇庆农商银行组建工作，引进发展金融机构6家以上，新增上市（挂牌）企业8家，金融业增加值增长10%以上。整合政府性资产，优化组建做实城投、交通、水务、金控、文旅五大投融资企业，全面抓好县级城投企业建设。全年完成100项市重点建设项目年度计划投资568亿元，产业项目投资增长63.7%。

　　二、深入推进“1133”工程，加大力度培育发展新动能

　　全力抓好“1133”工程落实。继续落实培育高新技术企业等“八大举措”，构建政策链、资金链、产业链、创新链深度融合的创新生态环境，努力创建国家创新型城市。高起点规划建设广东应用型高等教育基地，加强省市共建肇庆学院，争取广东工商职业学院获批升本，动工建设广东华商学院，大力支持肇庆医专扩容升本，加快推进华南农业大学珠江学院、广东工业大学华立学院等本科院校落户建设，争取再新引进1所高水平理工科大学，尽快推动高等教育规模质量上新水平。实施新型研发机构认定和扶持政策，新型研发机构增加到10家，规上工业企业建立研发机构占比提高到35%。大力推进高新区创新创业科学园、新区科技创新中心和端州创新科技园（市委党校旧址改建）等载体建设，引进建设大学科技园和研究院，力争高水平科技企业孵化器及众创空间总量16家，市级高新技术企业总量超过400家。以肇庆新区为依托筹建西江高新区，力争完成省级高新区申报认定。建立创新人才“绿卡”制度，力争新引育一批高层次人才。

　　加快肇庆高新区国家自创区建设。高标准参与珠三角国家自创区建设，尽快形成以高新区为核心区，以端州、鼎湖、高要、四会、新区等5个高新区分园为功能区，粤桂合作特别试验区、广佛肇（怀集）经济合作区等为拓展区的“1+5+N”国家自创区建设格局。高起点做好高新区220平方公里扩区调区规划建设，加快引进并推动与高校共建产业技术研究院，力争成功申报省级新型研发机构1家，高企总数120家、孵化器面积50万平方米。加大改革力度，推进园区更新管理和“智慧大旺”建设，抓好大部制行政管理体制改革。力争综合实力进入国家级高新区前90名。

　　打造肇庆科技创新产业带。充分利用粤港澳大湾区和广深科技创新走廊建设机遇，以高新区、新区和正在谋划的空港经济区为核心，实施连片发展，主动对接先进地区创新要素外溢转移，积极构建“北京、广深孵化—肇庆加速、肇庆落地”的创新产业链，打造粤港澳大湾区科技产业创新重要承载地。扎实推进国家知识产权示范城市培育建设，争创国家科技成果转移转化示范区。

　　三、深入推进新区崛起府城复兴，加快建设高品质新都市

　　高标准统筹推进新都市建设。立足肇庆山湖城江生态城市特质，坚持生态优先、绿色发展，注重展现浓厚历史文化底蕴，努力把肇庆建设成为依山环湖拥江的山水之城、历史名城、智慧新城。推动“一江两岸五区”同规同建同管，促进高要与端州、鼎湖与新区加快融合发展，引导农业人口向城市集聚，形成超百万人口城市发展格局。做好环星湖周边、东进大道沿线、北岭山麓和一江两岸的设计，控制好城区建筑高度、密度、风貌、容积率，抓好端州大冲片区、高要江滨新城等连片开发建设，着力把肇庆建设成为宜居宜业宜游新都市。

　　全力聚焦新区崛起。加快新区扩容提质步伐，完成480平方公里扩容规划修编工作，为创建国家级新区打好基础。加大基础设施建设力度，基本建成综合管廊、六大水系等基础设施，教育、体育、医疗等公共服务设施逐渐投入使用，加快东进大道、西江明珠等标志性工程建设，全力实施29项重点建设项目，完成年度计划投资112亿元，形成核心区城市框架。大力发展研发设计、文化创意、科技金融、云计算及大数据等都市型产业，打造大湾区智慧物流基地，着力把新区建设成为广东数字经济发展新高地。

　　聚焦府城复兴加快城市更新。持续推进府城保护与复兴项目，完成二期房屋征拆工作，抓好古城墙修缮保护，做好包公府衙和两广总督府设计，展现府城宋元明清的历史面貌和总体轮廓。建好城墙公园，加快崇禧塔片区改造，初步形成以府城为中心的城区新面貌。启动天宁北路段等城市更新项目和“肇庆亮化”工程，年内新开通“瓶颈路”18条，如期完成江滨堤路、河旁路等路网升级改造。抓好鼎湖区和高要城区更新工作，完善公共停车位等城市资源管理，增强城市运营水平和集聚能力。

　　推进县城扩容提质。高标准抓好县域新一轮城市总体规划编制，加快县城扩容提质建设，全面推进镇改街、村改居，着力解决部分县域“有县无城”问题。深入推进户籍制度改革，依托园区建设发展实体经济，鼓励外出务工人员和乡贤回乡就业创业，促进人口回流集聚。精心培育一批符合未来发展方向的特色小（城）镇。

　　四、紧抓粤港澳大湾区建设机遇，推进开放型经济发展

　　加快融入大湾区一体化发展。充分利用肇庆独特的区位条件、丰富的土地资源、明显的成本优势，着力打造大湾区连接大西南枢纽门户城市、科技产业创新重要承载地、新能源汽车与节能环保产业基地、康养旅游生态名城。全力做好与大湾区一体化交通对接，抢抓珠三角新干线机场规划建设机遇，动工建设肇明高速和水宾延长线，加快推进广佛肇高速二期、对接佛山11项路网工程和肇庆大桥扩建、国省道升级改造等项目，做好深江肇高铁、广湛高铁等前期工作，协调增加肇庆至深圳高铁班次，确保汕昆高速肇庆段、东进大道一二期通车，构建“十横八纵”交通大动脉，力促融入粤港澳大湾区一小时交通圈。

　　大力发展枢纽门户经济。推进粤港澳大湾区与大西南人流、物流、资金流、信息流在肇庆集聚辐射。依托深江肇高铁规划，谋划建设以肇庆东站为枢纽的大湾区大西南企业总部基地。以肇庆新港为核心构建“一市一港”“肇深协同”的港口物流新格局，发展一站式综合物流基地，打造辐射西南地区的水运集散中心。做强封开、怀集等“西联”门户经济，高起点规划建设粤港澳大湾区（怀集）绿色农副产品集散基地，不断提升粤桂合作特别试验区产业资源要素集聚能力。

　　提升对外开放合作水平。主动参与“一带一路”建设，切实在交通互联和产能合作等方面有所突破。打造深港先进制造产业园等吸收外资新平台，出台实施我市利用外资政策，提高吸引外资能力和利用外资水平。深化通关便利化改革，加快肇庆新区申报综合保税区，营造新区外向型经济发展新优势。推动外贸企业提高产品质量，力促进出口总额增速由负转正。加强与深圳等地产业深度融合，谋划与香港共建高端国际化康养基地，积极探索与港澳深打造“飞地经济”。加强与黑龙江鸡西市对口合作。

　　五、加大统筹协调和规划，激活城乡区域发展活力

　　实施乡村振兴战略。按照产业兴旺、生态宜居、乡风文明、治理有效、生活富裕的总要求，加快推进农村现代化。大力发展现代农业、乡村旅游、健康养生、中药材种植等相关产业，促进农村一二三产业融合发展。全面实施新农村建设书记工程，抓好各县（市、区）乡村振兴示范镇建设，并结合发展旅游、文化等产业，打造成为可看可学的示范样板镇。充分发挥村民主体作用，通过“财政补助一点、规划节省一点、成本降低一点、政策引导一点、乡贤支持一点”加大新农村建设投入，完成西北板块80%、东南板块全部自然村人居环境综合整治任务。省定贫困村全部行政村年底前达到整治标准。高起点抓好四会市全域推进社会主义新农村建设示范县。推进“厕所革命”，加快补齐影响农村生活品质突出短板。深化农村综合改革，完成农村土地承包经营权确权登记颁证任务，抓好中央扶持村级集体经济发展试点、省级农村集体产权制度改革试点，创新农村普惠金融服务。加快推进乡村治理体系和治理能力现代化。

　　引导县域做强主导产业。加强对西北板块的统筹支持，推动东南板块通过联合招商、产业共建、对口帮扶等形式带动西北板块发展。引导支持县域发展特色主导产业，支持怀集食品加工、德庆先进装配式建筑、封开新型建材、广宁高端生态旅游康养等特色主导产业发展。打通山区致富路，加快“四好农村路”建设，推进山区县出境省道拓宽改造，加快实现全部乡镇半小时上高速公路。推进农业供给侧结构性改革，稳定粮食生产，新建高标准基本农田19.5万亩，加快推进国家级（德庆）南方道地药材批发市场等项目，谋划建设农业科技园区，促进农村农业提质发展。

　　坚决打好精准脱贫攻坚战。进一步压实脱贫攻坚责任，建立长效脱贫机制，做到严要求、真感情、看长远、做实事。积极筹建就业引导基金，鼓励贫困户外出就业，把扶贫和扶志、扶智紧密结合起来。加强扶贫专项资金严格监管和县级统筹使用，发挥资金最大效益，集中力量发展固定回报收益好的产业，拓宽稳定脱贫途径。全面落实“1+N”扶贫政策和义务教育、基本医疗、住房安全三大保障，加强政策性保障兜底，确保今年再实现2.3万贫困人口预脱贫，按中央和省要求如期打赢精准脱贫攻坚战。积极落实与广西贺州市、桂林市对口协作工作。

　　六、着力推进“绿富同兴”，加快创建国家生态文明建设示范市

　　坚决打好污染防治攻坚战。认真抓好中央和省环保督察反馈问题整改落实，全面深入开展“消烟、清江、净土”行动，下大决心解决突出环境问题。狠抓工业源整治和扬尘源监管，严管整治瓷土开采，不新增瓷土开采，严打盗采超采，到期不再续期，从源头上减少陶瓷行业污染排放。开展西江船舶大气污染专项整治，强化机动车和施工机械尾气排放整治，力争城区空气优良率达90%左右，空气质量综合指数全省排名再上升。强化落实市县镇村四级河长分级责任制，严格实施湖长制，强化北岭山违法用水清理，压实星湖污染防治责任，接通端州城区断头污水管网，不让污水进入星湖。加强“一河一策”治理，抓好黑臭水体专项整治，依法依规清理违法禽畜养殖场和饮用水源保护区违法项目，开展端州城区垃圾分类减量试点，启动建设城镇污水处理厂62座，加强雨污分流管网建设，提高城镇污水处理率。扎实推进土壤污染防治行动计划，摸查土壤环境质量状况，基本建立土壤环境监管体系，抓好一批土壤治理修复试点。

　　推进绿水青山与金山银山共赢发展。充分挖掘依山傍水自然优势，支持发展高端康养医药产业，探索形成大旅游绿色发展模式。加强自然保护区管理。扎实推进绿化肇庆大行动，抓好生态公益林建设和林分改造，新增森林公园2个，建设森林碳汇重点生态工程2.93万亩、生态景观林带完善提升工程1618亩。绿化美化乡村109条，新增一批省级生态镇（乡），推进森林小镇建设。

　　推进资源节约和循环利用。加强土地节约集约利用，保持高压整治“三违四抢”，严守耕地保护红线。深入整治非法采运河砂等矿产资源。加快工业园区集中供热项目建设，实施水泥、陶瓷、金属加工等重点行业节能降耗，推广应用先进适用清洁生产技术、新能源公交车和汽车。

　　七、全力办好省运会，坚持不懈创建全国文明城市

　　把第15届省运会办成一流盛会。落实省运会组委会工作部署，确保3月底前完成34个体育场馆工程建设任务。全面完善相关路网、市政、通讯、无障碍设施等配套建设。精心组织开、闭幕式，展现肇庆精气神。建立高效畅通的指挥协调系统，织密安保网，完善交通、食品卫生、医疗救护等应急保障体系，高标准做好接待、宣传、志愿服务等工作。抓好竞赛组织和备战训练，努力实现办会、参赛双丰收。将举办省运会与“创文”、星湖景区创5A、城市提质有机结合起来，推动城市建设、管理水平、文明素质上新台阶。

　　全面推进新一轮文明创建。坚持“创文”决心不变、劲头不减，建立健全长效工作机制。全力推动“创文”向山区县城、镇村和基层单位持续深入，推动全域人居环境改造、素质提升的文明建设。全面完成城区老旧小区改造任务，推进城区背街小巷升级改造，加快补齐市场管理、社区公共文化设施建设等短板，让群众有更多“创文”获得感，争取年度测评成绩进入全国前列。

　　依托省运会加快大文体旅产业融合发展。充分发挥省运会的拉动作用，促进体育与旅游、文化、健康产业融合发展，培育“体育+”“旅游+”经济。深度挖掘肇庆历史文化资源，凝练提升区域文化特色，推动叶挺独立团团部旧址等申报国家级文物保护单位。围绕影院建设、文化创意、演艺娱乐等领域发展文体产业，提高文体旅产业对全市经济发展的贡献率。

　　八、坚持以人民为中心，提高保障和改善民生水平

　　狠抓教育、医疗、公共交通三大民生服务。启动实施教育提升行动计划，全面提升教育现代化水平，提前谋划城区幼儿园、中小学校布局的规划调整，新建扩建义务教育学校9所、新增学位10000余个，持续解决城镇优质学位不足和大班额等问题。狠抓强师振教工程，构建教育教学质量保障体系，尽快扭转生源外流情况。支持发展民办优质教育，提升对人才落户的吸引力。办好继续教育，提升老年教育。启动实施卫生与健康提升行动计划，全面深化医药卫生体制综合改革，建立健全群众满意的医疗服务体系，补强医疗卫生基础设施短板，加快医院升级建设，新增床位3500个，探索实施与高水平医院共建共管新模式，提升群众本地就医服务水平。与广州中医药大学合作推进中医药强市建设。确保通过国家卫生城市复审。优先发展城市公共交通，加大投入实施“四大畅通”工程，优化城区公交站场、线路、班次设置，切实提高城市公交服务水平。

　　持续提高就业、社保、住房等民生保障水平。促进多渠道就业创业，新增城镇就业4.25万人、城镇失业人员再就业1.25万人。基本养老保险参保率提高到88%以上，企业职工基本养老保险参保率和缴费率均达到省下达的任务要求，确保社保基金平稳有序运行。切实维护和谐的劳动关系。全面提高城乡低保补差水平，城乡特困人员供养标准达到当地低保标准的1.6倍，城乡困难群众医疗救助比例达到80%。推行养老服务机构公建民营、医养结合试点，加快建设复退军人服务体系。开工建设棚户区改造住房2811套，基本建成保障性住房、棚户区改造住房3786套。建成乡镇干部周转房2511套。

　　持续推进“平安肇庆”建设。加强和创新社会治理，完善社会矛盾排查预警调解和社会稳定风险评估机制，进一步畅通信访渠道，及时解决群众合理诉求，用法治思维和方式预防化解社会矛盾。深入实施“扫黄打非”专项行动，依法严厉打击各种违法犯罪活动。加强安全生产和食品药品安全监管，铁腕整治各类风险隐患，坚决遏制安全事故。维护市场交易和质量安全。统筹防灾减灾救灾工作。

　　与此同时，支持国防和军队改革，加强全民国防教育和国防动员建设，推进军民融合和“双拥”工作，提升国防后备力量建设质量。继续办实十件惠民实事（详见附件一）。做好统计、民族、宗教、革命老区、外事、侨务、港澳、对台、人防、地方志、档案、气象、水文、地震、妇女儿童、青少年、老龄、残疾人、慈善等工作。

　　九、切实提高政府运作水平，塑造良好营商发展环境

　　坚持依法行政，打造法治政府。自觉接受人大监督，落实市政府重大决策出台前向市人大常委会报告制度，支持市政协履行政治协商、民主监督、参政议政职能，定期向人大及其常委会报告工作和向政协通报情况。主动加强与各民主党派、工商联、无党派人士和各人民团体的沟通协商。坚持严格依法办事，落实政府法律顾问制度，切实做到法定职责必须为、法无授权不可为，推动政府工作全面纳入法治轨道，确保创建成为珠三角法治政府示范区。

　　坚持以民为本，打造服务政府。增强群众观念和群众感情，全面推行服务承诺、限时办结，使政府服务更加精准高效。推进“减证便民”行动，大力规范中介服务，深入清理整合中介事项，完善中介事项清单管理。扩建市行政服务中心，强化服务职能，构建信息资源共享平台，形成集中统一的为民办事平台，把面向企业群众的服务事项纳入“1+N”实体大厅，拓展“零跑动”服务，实现“一个中心、一个窗口、一张网络、一次办成”。

　　坚持改革创新，打造效率政府。坚持用珠三角标准和大湾区意识推动工作，坚持“马上就办、办就办好”要求，发扬钉钉子精神，进一步加强政府职能部门“统筹、协调、指导、监督”意识和能力，推行重点工作会商制度，着力提升政府办事效率。强化牵头单位主体责任，建立“谁牵头、谁负责，谁协助、谁配合”的联动工作机制。深入推进“放管服”改革，加大简政放权力度，推进省以下财政事权和支出责任划分改革，优化市、区两级行政权限设置，增强基层执行力。深化商事登记“多证合一”“证照分离”改革，全面推进“双随机一公开”监管，建成行政审批效能监管监督系统平台，进一步减少投资项目审批事项。大力推进“数字政府”建设，加快搭建统一安全的政务云平台，促进数据共享，优化政务流程，切实提高政府运作效能。

　　坚持从严治政，打造廉洁政府。认真开展“不忘初心、牢记使命”主题教育。严格落实全面从严治党要求，履行主体责任，把党风廉政建设引向深入。落实中央八项规定精神及实施细则，驰而不息纠正“四风”。加强对行政权力运行的制约监督，把权力关进制度的笼子。落实容错纠错预警机制，大力整治庸政懒政怠政和不作为、乱作为。全面配合推进监察体制改革试点工作。强化对国家和省重大政策落实、财政资金、国有资产、公共资源、政府重点投资项目、新农村建设以及领导干部履行经济责任的审计监督。着力解决损害群众利益的不正之风和腐败问题，构建“亲”“清”新型政商关系。

　　各位代表，新时代新肇庆，新气象新作为!让我们紧密团结在以习近平同志为核心的党中央周围，按照省委、省政府和市委的决策部署，不忘初心，牢记使命，解放思想，实干兴肇，为全面建成小康社会、加快建设广东面向大西南枢纽门户城市而努力奋斗！为把广东建设成为向世界展示习近平新时代中国特色社会主义思想的重要“窗口”和“示范区”作出肇庆更大贡献！
